# Dental pulp stem cells retain mesenchymal phenotype despite differentiation toward retinal neuronal fate *in vitro*

**DOI:** 10.3389/fmed.2022.821361

**Published:** 2022-10-12

**Authors:** Aishwarya Balasankar, Shu-Yi Claire Chan, Venkata Pakala Sudheer Babu, Gary Yam, Goh Bee Tin, Shweta Singhal

**Affiliations:** ^1^Singapore Eye Research Institute, Singapore, Singapore; ^2^National Dental Centre Singapore, Singapore, Singapore; ^3^Singapore National Eye Centre, Singapore, Singapore; ^4^Duke NUS Medical School, Singapore, Singapore

**Keywords:** dental pulp stem cells, mesenchymal stem cells, retinal ganglion cells, retinal differentiation, retinal stem cell therapy

## Abstract

Dental pulp stem cells (DPSCs) are an easily accessible, heterogenous source of mesenchymal stem cells (MSCs) that are derived from the neural crest. Evidence suggests that they have neurotrophic qualities in their undifferentiated state and can also be differentiated into neuronal and retinal cell types. There is growing interest in using DPSCs in cell-based therapies to treat glaucoma and blinding retinal diseases. However, careful characterization of these cells is necessary as direct intravitreal and subretinal MSC transplantation is known to lead to deleterious glial reaction and fibrosis. In this study, we provide evidence for the mesenchymal-predominant nature of DPSCs and show that DPSCs maintain their mesenchymal phenotype despite upregulating mature retinal markers under retinal differentiation conditions. CD56, which was previously thought to be a specific marker of neural crest lineage, is robustly co-expressed with mesenchymal markers and may not be adequate for isolating a subpopulation of neural crest cells in DPSCs. Therefore, identification of more specific markers is required to elucidate the heterogeneity of the population and to successfully isolate a putative neural stem cell population before DPSCs can be used for retinal therapy.

## Introduction

In the last decade, there have been major advances in stem cell therapy for blinding retinal diseases like age-related macular degeneration. In addition to the use of embryonic and induced pluripotent stem cells, there has been significant interest in the use of adult mesenchymal stem cells (MSCs) from other sources such as bone marrow, cord blood, adipose tissue and muscle. These cells are available in abundance and can be isolated from the patient autologously, overcoming problems of immune rejection.

The applications of MSC therapies for eye disease have expanded in the last decade. MSCs exert a significant paracrine effect and many groups across the world have attempted transplantation of undifferentiated MSCs in the subretinal or intravitreal space for neuroprotection. Although results have been promising, particularly in glaucoma and optic nerve crush models (1, 2), there is considerable evidence that intravitreal transplantation of MSCs can induce extensive reactive gliosis (3), severe vitreoretinopathy and epiretinal membrane formation (4). In the human eye which has a larger vitreous space compared to rodent eyes, these effects are more pronounced. Attempts at MSC transplantation into the subretinal space in humans have led to epiretinal membrane formation, proliferative vitreoretinopathy and even tractional retinal detachment (5–8). Transplantation of stem cells of mesenchymal origin for retinal therapy must therefore be undertaken with extreme caution.

Adult MSCs are also being evaluated as autologous sources of stem cells for retinal therapy. When subjected to differentiating conditions *in vitro*, these cells have been shown to acquire retinal cell markers and to a limited extent, even function like retinal neurons (9). However, not all cells undergo this differentiation. In many cases it is not clear what proportion of cells have differentiated into retinal neuronal precursors. It is also not known if these ‘retinal’ cells still possess mesenchymal properties. Given the high risk of mesenchymal cell associated blinding complications in the subretinal and intravitreal space, it is imperative that MSC-derived retinal cells being considered for clinical use be carefully evaluated for their mesenchymal phenotype at the end of the differentiation process.

Dental pulp stem cells (DPSCs) are a population of MSCs derived from adult human teeth. They contain a mix of different stem cell types including mesenchymal, vascular and neuronal stem cells (10). Like other adult MSCs, DPSCs have been shown to demonstrate a paracrine neurotrophic effect (11, 12). *In vitro*, they also possess the ability to upregulate expression of neuronal markers in general (13) and markers of retinal cell fates like photoreceptor and retinal ganglion cells in particular (9, 14). It has been suggested that DPSCs may be used as a source of stem cells for retinal cell replacement therapy. In this study, we established primary DPSC cultures, evaluated them at baseline, and directed them toward a retinal fate with particular focus on their mesenchymal phenotype during and at the end of differentiation.

## Materials and methods

### Isolation and culture of primary human dental pulp stem cells

Dental pulp stem cells isolation and culture was performed as previously described (10). Healthy non-infected human teeth (molar, premolar and canine) were collected with informed consent from adult patients (15-41 years of age) undergoing routine extraction for orthodontic reasons, under approved guidelines set by the Centralized Institutional Review Board (SingHealth Research). Teeth were cleaned in iodine solution and cracked open using a power drill to expose the pulp chamber. Dental pulp was extracted with forceps, minced with a scalpel and digested in a solution of 3mg/ml collagenase and 4mg/ml dispase (Roche) for 1 h at 37°C. Single-cell suspensions were obtained by straining the cells through a 70 μm cell strainer (Falcon). Dental pulp cell suspensions were seeded at a maximum density of 1,100 cells per cm2 in [growth medium] and maintained at 37°C and 5% CO2.

### Induction of retinal differentiation

phDPSCs were induced toward a photoreceptor fate as described previously (15). Briefly, cells were seeded on laminin-coated plates, allowed to expand for three days before being switched to Neurobasal A media (Gibco, Thermo Fisher Scientific) supplemented with B27 (1:50, Thermo Fisher Scientific), epidermal growth factor (20 ng/mL, Sigma-Aldrich) and basic fibroblast growth factor (40ng/mL, Sigma-Aldrich). On day 8 of differentiation, this was changed to 50% DMEM (Gibco, Thermo Fisher Scientific) + 50% F12 (Gibco, Thermo Fisher Scientific) media supplemented with insulin-transferrin-sodium selenite (conc, Gibco, Thermo Fisher Scientific) and basic fibroblast growth factor (40 ng/mL, Sigma-Aldrich). From day 15 to day 21 of differentiation, retinoic acid (0.5 μg/ml, Sigma-Aldrich) was added to the culture medium. All differentiation media contained penicillin (50 μg/ml, Gibco, Thermo Fisher Scientific) and streptomycin (500 μg/ml, Gibco, Thermo Fisher Scientific) to prevent bacterial contamination. Cells were photographed/lysed to isolate RNA or fixed for immune-cytochemistry at weekly time points.

### Fluorescence-activated cell sorting analysis

Pre-conjugated primary antibodies to CD56 and CD15, CD24, CD29, CD90, CD34, and CD45 (Supplementary Table 1) were used to label the DPSC cells in suspension as per manufacturer’s instructions; along with Propidium iodide (Molecular Probes, Thermo Fisher Scientific) to discriminate live cells. Stained cells were analyzed on the BD FACSverse 3L8C fluorescence-activated cell sorter and data analysis was carried out using BD FACS suite V1.0.6. For list of antibodies used see Supplementary Table 1.

### mRNA expression analysis

Total cellular RNA was extracted from cell pellets using the Pico Pure RNA isolation kit (Thermo Fisher Scientific). 1μg of total RNA was reverse transcribed using the iScript Reverse Transcription Supermix for RT-qPCR system (Bio-Rad) in a C1000 Thermal Cycler (Bio-Rad). Real-time RT-PCR was carried out on 2μl of the synthesized cDNA and 0.5μl each of forward and reverse primers 20μM, using Bio-Rad SYBR Green Master Mix (containing dNTPs, MgCl2, and DNA polymerase). For list of primers used see Supplementary Table 2.

### Immunocytochemistry

Adherent cells on coverslips in culture were washed thrice in DPBS and fixed in 4% PFA for 10 minutes, followed by incubation in blocking buffer (DPBS, 1% BSA, 100mM Glycine, 0.3% Triton X-100) for 60 min. Immunostaining was performed in antibody dilution buffer (DPBS, 1% BSA, 0.3% Triton X-100) overnight at 4°C. Goat anti -OTX2 (Novus biologicals AF1979); rabbit anti-Rax (ab23340 Abcam) or mouse anti MITF (Millipore MABE78) was used along with FITC conjugated CD90. Secondary antibodies conjugated with Alexa Fluor (Invitrogen) were diluted 1:1000 in antibody dilution buffer and incubated for 60 min at room temperature. Coverslips were mounted on a microscopic slide using Antifade Mounting Medium. Image acquisition and processing were performed using AxioVision software (Zeiss).

## Results

### Undifferentiated phDPSCs are predominantly mesenchymal in nature

**FIGURE 1:**
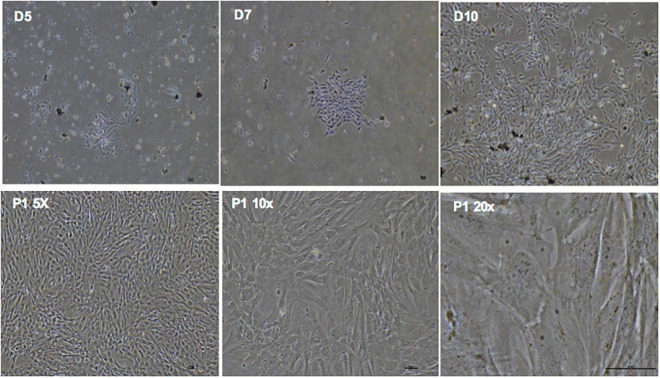
Primary human dental pulp stem cells (DPSC) show fibroblastic morphology in culture.(D5, D7, D10) Phase contrast microscopy images in the **top panel** show clonal expansion of DPSC in primary culture with expansion to confluence over time. **Bottom panel** shows confluent DPSC at increasing magnification in subsequent passages also showing predominantly fibroblastic morphology. Scale bar = 50 μm.

phDPSC cultures were established from a total of 44 teeth collected from 30 donors. In each case cell colonies first appeared after 5-10 days in culture and grew into confluent monolayers by week 3. phDPSCs could then be cultured up to passage 9, cryopreserved and revived from cryopreservation without change in morphology or loss of proliferative ability. In culture, phDPSCs demonstrate a fibroblastic morphology (Figure 1).

**FIGURE 2:**
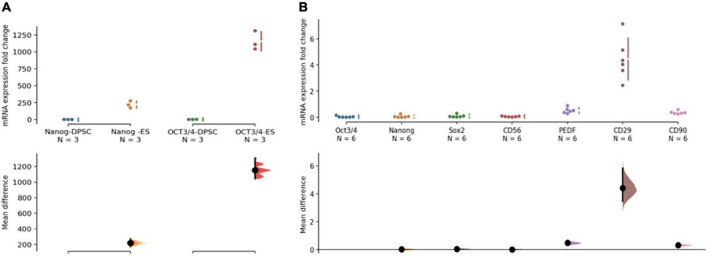
Primary human dental pulp stem cells (DPSC) are predominantly mesenchymal in nature. **(A)** DPSC express pluripotency markers Nanog and OCT 3/4 at 100-1000 fold lower levels compared to human embryonic stem cells (hESC). The quantitative PCR analysis was performed on mRNA from 3 different primary DPSC lines and compared against different replicates from one HESC line. **(B)** Primary DPSC express higher levels of mesenchymal markers (PEDF/CD29 and CD90) compared to pluripotency markers (Oct 3/4, Nanog and Sox2). CD56 expression is seen at low levels equivalent to that of Oct 3/4. The analysis was performed on cells from 3 different cell lines and 2 different passages with a total *n* = 6. In both graphs, raw data and spread is shown as a Cumming estimation plot on the upper axes and mean difference compared to control sample are plotted as bootstrap sampling distributions on the lower axes. Each mean difference is depicted as a dot and each 95% confidence interval is indicated by the ends of the vertical error bars.

Although phDPSCs express pluripotency markers Oct 3/4 and Nanog, the level of mRNA expression compared to human embryonic stem cells is 100-1000 fold lower (Figure 2A). Compared to pluripotency markers, phDPSC expression of mesenchymal markers CD90, CD29 and PEDF is significantly higher (Figure 2B).

**FIGURE 3:**
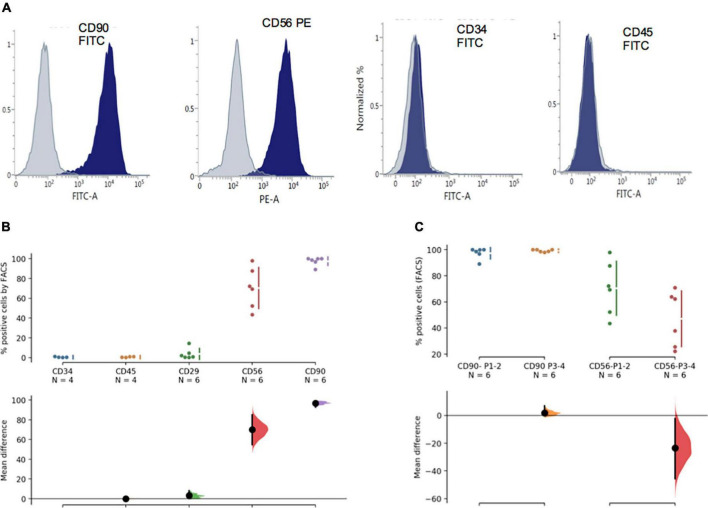
PhDPSC analysis immunophenotyping by FACS shows predominant CD90 expression. **(A)** Top panel shows representative FACS histograms for each of the markers tested including hematopoetic markers CD 34 and CD45, mesenchymal markers CD29 and CD90 as well as neural crest marker CD56. Gray plot shows negative control and blue plot shows positive population. **(B)** Quantitative analysis by FACS is depicted as percentage positive cells and shows negligible expression of CD34 and CD45, slightly higher percentage of cells express CD29 with significantly higher percentage expressing CD 56 and CD 90. The analysis depicts pooled data from experiments performed on 2-3 different cell lines from passage 1-2. CD34 expression is used as the standard against which remaining samples are compared. **(C)** CD90 and CD56 percentage positive cells in primary DPSC is depicted over time in culture. Despite repeat passaging, percentage of CD90 cells remain high and stable in the phDPSC population. A trend toward decline in percentage of CD56 cells with passage is seen although the mean difference between early and later passages is not significant. CD 56 expression at each passage also shows significant variability (as seen by the spread of the individual data points). This analysis was performed on cells from 3 different cell lines comparing expression in P1-2 with P3-4. In **(B,C)**, raw data and spread is shown as a Cumming estimation plot on the upper axes and mean difference compared to control sample (In B, CD34 data is used as control) are plotted as bootstrap sampling distributions on the lower axes. Each mean difference is depicted as a dot and each 95% confidence interval is indicated by the ends of the vertical error bars.

Flow cytometric analysis shows that over 95% of the phDPSC population is positive for CD90 with 50-80% positive for CD56 (Figures 3A,B). The high proportion of CD90 positive cells is maintained in culture despite repeated passage (Figure 3C). Similarly the size of the CD56 positive cell population within the phDPSC does not show significant change with passage although there is greater individual fluctuation between cell lines. The variability in proportion of CD90 and CD56 positive cells in different primary DPSC cell line is shown in Supplementary Figure 1 and suggests that there cells derived from different donors may have different stem cell potential. As reported previously (16), phDPSC show negligible expression of hematopoietic stem cell markers CD34 and CD45.

### phDPSCs retain a mesenchymal phenotype despite differentiation toward retinal fate

**FIGURE 4:**
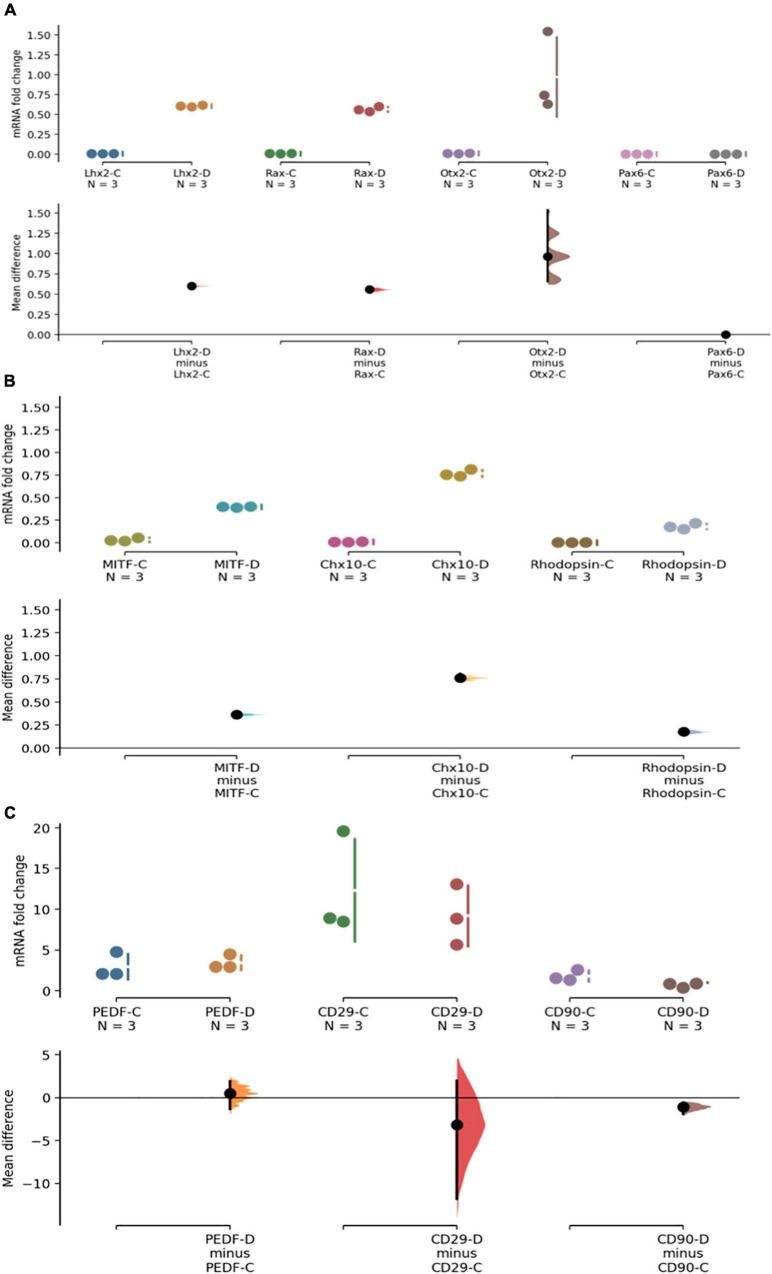
PhDPSC cultured in retinal differentiation conditions –D continue to express high levels of mesenchymal markers despite acquisition of retinal progenitor and retinal subtype specific precursor markers. **(A)** Top panel shows up-regulation of mRNA expression of retinal progenitor markers LHX2, RAX, OTX-2, and PAX6 in primary DPSC cultured in retinal differentiation conditions –D compared to primary DPSC cultured in control conditions –C after 4-6 weeks in culture. Note that no expression of PAX6 is seen in either control or differentiated cells. **(B)** Middle panel shows a small but significant upregulation of retinal subtype-specific precursor markers MITF, CHX-10, and Rhodopsin in phDPSC cultured in retinal differentiation conditions –D compared to the same cells cultured in control conditions –C after 4-6 weeks in culture. **(C)** Bottom panel shows that there is no significant decline in levels of mesenchymal markers PEDR, CD29 and a small decline in CD90 between control –C and differentiated –D phDPSC. Also note that the fold change values on the *Y* axis are very small in **(A,B)** while being significantly higher in **(C)** indicating that acquisition of retinal markers is minimal while expression of mesenchymal markers remains robust despite the differentiating conditions. In all the above graphs, raw data and spread is shown as a Cumming estimation plot on the upper axes and mean difference compared to each sample’s control are plotted as bootstrap sampling distributions on the lower axes. Each mean difference is depicted as a dot and each 95% confidence interval is indicated by the ends of the vertical error bars.

It is known that a small number of DPSCs can acquire retinal markers when differentiated toward a retinal fate in animal models. Given our data showing the predominantly mesenchymal nature of phDPSC, we evaluated the fate of these cells when placed in retinal differentiating conditions *in vitro*. Specifically we evaluated the mesenchymal phenotype of DPSC cells that acquire retinal markers. We find that phDPSC placed in retinal differentiation conditions upregulate mRNA expression of early eye field transcription factors such as LHX2, RAX and OTX2 (Figure 4A) and retinal precursor markers such as MITF, CHX10 and Rhodopsin (Figure 4B). However, despite acquisition of these retinal markers, phDPSC continue to concurrently express significant mRNA levels of mesenchymal markers PEDF, CD29 and CD90 (Figure 4C).

**FIGURE 5:**
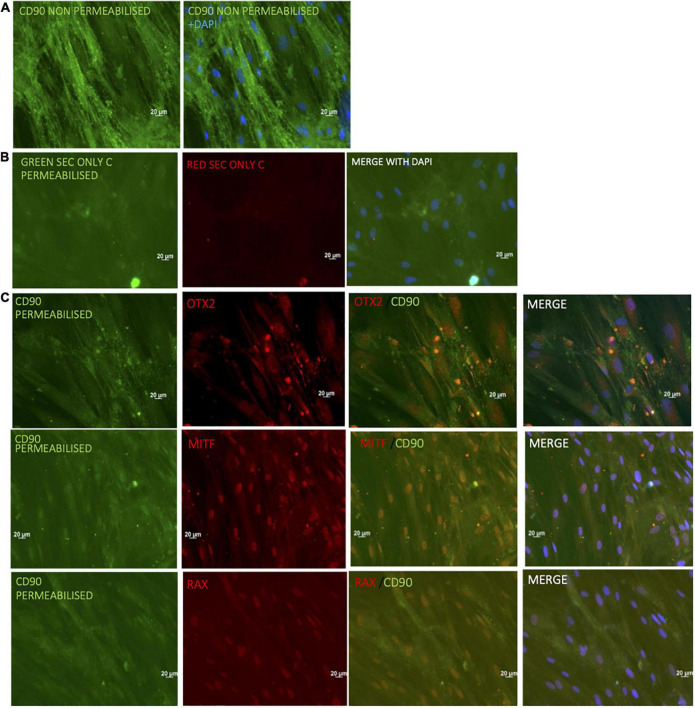
Immunocytochemistry confirms CD90 expression in phDPSC acquiring retinal progenitor markers. Robust CD90 expression (green) is seen in untreated phDPSC cells by immunocytochemistry. **(A)** This panel shows signal strength when ICC is performed in the absence of permeabilization (to preserve the signal of cell surface marker CD90). **(B)** These panels show secondary only controls for red and green signals when ICC is performed with permeabilization to detect nuclear markers. **(C)** Expression of CD90 and early eye field transcription factors OTX2, MITF, and RAX in phDPSC treated to retinal differentiation conditions after 50 days in culture. The CD90 signal is quenched compared to A due to the permeabilization required to elicit signal from nuclear antigens OTX2, MITF, and RAX but can be compared to the secondary only control in B to assess the positivity of the signal. Despite the quenched signal, CD90 (green) is seen not only in control cells but in treated cells colocalizing with nuclear expression of OTX2, MITF, and RAX.

Evaluation of CD90 expression of phDPSC undergoing retinal differentiation by immunocytochemistry (ICC) confirmed that cells acquiring retinal markers continued to express CD90 at levels comparable to undifferentiated cells. Note that to preserve expression of the cell-surface marker CD90, ICC is usually performed without permeabilization. When phDPSC are labeled without permeabilization, this results in a robust CD90 signal (Figure 5A). However, to assess colocalization of CD90 with eye field transcription factors like OTX2, MITF and RAX which are expressed in the nucleus, the permeabilization step during ICC becomes necessary and results in quenching of the CD90 surface signal. This is evident in Figure 5B control cells where the CD90 signal is significantly dimmer compared to Figure 5A. Despite the quenched signal, CD90 expression is seen in the differentiating DPSC with co-localization of CD90 with retinal markers OTX2, MITF and RAX (Figure 5C). This suggests that even in retinal differentiating conditions, despite acquisition of early retinal progenitor markers, the phDPSC cells retain a predominantly mesenchymal phenotype.

### CD56 + phDPSCs are predominantly mesenchymal

Although CD56 was previously considered a neural crest marker, its specificity has come under question (17). To address this in the context of phDPSCs, we sorted these cells into CD56 + and CF56– subpopulations for further analysis.

**FIGURE 6:**
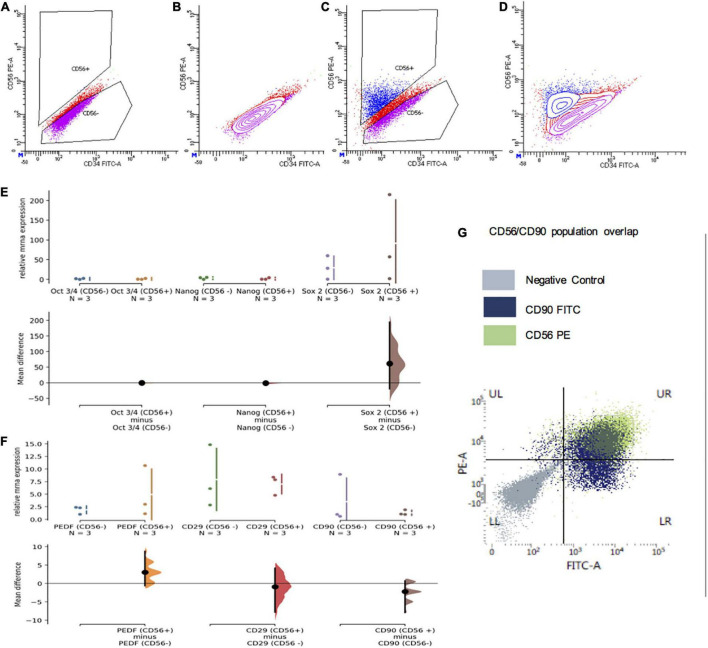
CD56+ subpopulations of primary human DPSC are also predominantly mesenchymal. **(A–D)** Representative flow cytometry plots demonstrating gating of CD56 negative and positive cells which was used to segregate primary DPSC into CD56+ and CD56- cells. **(A,B)** Scatter plots of primary DPSC cells stained with secondary (PE) only negative controls. **(C,D)** Scatter plots of cells stained with PE-conjugated CD56 antibody. **(E)** There was no significant difference in relative mRNA expression of pluripotency markers Oct 3/4, Nanog and Sox2 in CD56+ compared to CD56 cells. **(F)** There was no significant difference in relative mRNA expression of mesenchymal markers PEDF/CD29 and CD90 in CD56+ compared to CD56 cells. **(G)** FACS scatter plot showing significant overlap of CD56+ (green) and CD90+ (blue) cells in UR quadrant with most of the CD56+ cells showing positivity for CD90 as well.

Despite extensive optimization of settings, flow sorting of phDPSCs labeled with CD56 showed a continuous scatter plot without a distinct segregation between CD56 positive and negative populations. Gating was therefore performed using negative controls as a guide, with bias toward under-sorting to reduce likelihood of cross-contamination (Figures 6A–D).

Apart from there being no significant difference in cell morphology of the two groups in culture (data not shown), mRNA expression of pluripotency markers Oct3/4, Nanog and Sox2 was equivalent and negligible in both CD56 + and CD56− cells (Figure 6E).

Although there was apparent greater expression of SOX2 among the CD56 + cell samples, the mean difference remained statistically insignificant. In addition, not only did the CD56 + cells show expression of mesenchymal markers PEDF, CD29 and CD90, the expression level was equivalent to that seen in CD56– cells (Figure 6F).

FACS analysis of phDPSCs double-stained with CD56 and CD90 showed that most of the CD56 + cells were also positive for CD90 (Figure 6G). This was seen consistently across the different phDPSC cell lines and through passage 1-5 (data not shown).

Therefore, both CD56 + and CD56– phDPSCs were equivalent in terms of their expression of mesenchymal markers at both a population and single-cell level, with most CD56 + phDPSCs co-expressing mesenchymal marker CD90.

## Discussion

In this study, we present evidence that phDPSCs are a predominantly mesenchymal population. Our data suggests that not only are phDPSCs highly mesenchymal in their undifferentiated state, they maintain a mesenchymal phenotype despite attempted differentiation toward a retinal fate. We demonstrate that this holds true at both a population level (where results can be confounded by different cell types if the population is heterogenous) and at the level of the individual cell.

Compared to MSCs derived from other sources, DPSCs are thought to be embryologically derived from neurectoderm. Previously, the abundant presence of CD56 in DPSCs has been used to support a neuroectodermal origin (16, 18), suggesting that these represent a population of neural crest-derived stem cells persisting in the adult dental pulp niche. However, CD56 seems to be variably expressed in phDPSCs with no distinct segregation between strongly positive and negative cells. There is also significant variability in CD56 expression between different donor cell lines suggesting that the stem cell potential of DPSC derived from different donors might be different.

Expression of mesenchymal markers was equivalent in CD56− and CD56 + cells with both groups appearing fibroblastic in morphology and expressing equivalent and negligible levels of pluripotency genes. Expression of CD90 and CD56, particularly the former, was robust in culture despite repeated passage. Taken together, these results indicate that CD56 may not be the most specific marker of neurectodermal lineage and CD56 + cells do not represent a separate lineage from the mesenchymal-predominant phDPSCs. Our findings concur with previous studies which have also found high (though variable) proportions of CD56 in cultures of phDPSCs, as well as subpopulations of phDPSCs co-expressing CD56 together with MSC markers such as MSCA-1 (18, 19) and CD90 (16).

The use of CD56 as a reliable neural crest stem cell marker is being questioned in other MSC populations as well. Although initially thought to be absent from human bone marrow-derived MSCs (hBM-MSCs), hBM-MSCs have been shown to express CD56 and at variable proportions ranging from 25 to 90% (17) similar to our findings in phDPSCs. Rather than being a marker of neural stem cells, CD56 may instead be a more generic MSC marker. CD56 has long been known to be expressed widely in undifferentiated mesenchymal cells during human embryogenesis (20). CD56 is also not specific for cells of neuronal potential, being a marker of myogenic progenitor cells (21) including craniofacial muscle-derived stem cells with osteogenic, adipogenic, and myogenic potential (22). In hBM-MSCs, cells co-expressing both CD56 and the MSC marker MSCA-1 and CD56 represent a subgroup of MSCs with potent chondrogenicity (23). Therefore, CD56 may be either an MSC marker or a marker for a more-differentiated subgroup of phDPSC cells, committed to neurogenic or myogenic fates.

Previous studies have demonstrated the neurogenic potential of DPSCs both *in vitro* (24–26) and *in vivo* (24). Specifically, the ability of phDPSCs to acquire retinal markers *in vitro* has previously been shown in a variety of culture conditions, including in response to conditioned media from injured rat retinae (14) and in directed differentiation in 3D culture (9). Our data shows similar acquisition of early eye field transcription markers (including Rho, MITF and Chx10) by phDPSC. Like previous studies this happens within 2-4 weeks of directed differentiation. However at a single-cell level, the same cells which acquire markers of early eye field transcription also show robust expression of mesenchymal marker CD90. It is quite likely that the differentiation toward retinal cell fate is incomplete at this point. The persistence of mesenchymal markers at this stage therefore indicates that phDPSC are likely still predominantly mesenchymal despite acquiring retinal markers.

Careful characterization of MSC-derived cells is necessary if they are to be used in cell-based applications, as transplantation of inadequately differentiated MSCs into the subretinal or intravitreal space is known to cause extensive reactive gliosis (27). The hallmark of reactive gliosis is upregulation of vimentin (Vim) and glial fibrillary acidic protein (GFAP). Previous attempts at *in vivo* transplantation of DPSCs are consistent with development of reactive gliosis in the transplant. By culturing DPSCs in neural stem cell (NSC) media, Jung et al. (28) attempted to enrich the neural stem cell (NSC) subpopulation before transplantation into the rat central nervous system. However, while the NSC-cultured cells expressed higher levels of neural crest markers, they continued to express high levels of mesenchymal markers CD29 and vimentin. Moreover, high GFAP levels were found in the rat CNS after transplantation. Another key study by Mead et al. (1) showed that intravitreally transplanted DPSCs promote survival of neurons in crushed rat optic nerves. However, in rat eyes transplanted with live DPSCs, a strong activation of glial response was seen with robust expression of GFAP in the ganglion cell layer. These results support not only the mesenchymal-predominant nature of DPSCs but also their propensity for inducing reactive gliosis when transplanted into the central nervous system.

In conclusion, from the immunoprofiling of undifferentiated phDPSCs, the high expression of mesenchymal markers in the CD56 + population and the retention of mesenchymal markers in phDPSC undergoing retinal differentiation, it is clear that the phDPSC population is mesenchymal predominant. If there is a subpopulation of neural stem cells present we do not currently have the correct markers to identify them and use them for differentiation purposes. Since the DPSCs behave to be a mesenchymal predominant population, at this juncture, it is currently unsafe to use them as a source of retinal stem cells for *in vivo* therapy of retinal eye diseases. Identification of more specific markers is required to elucidate the heterogeneity of the population and to successfully isolate the neural stem cell population from the phDPSCs before the cells can be used for retinal therapy.

## Data availability statement

The raw data supporting the conclusions of this article will be made available by the authors, without undue reservation.

## Ethics statement

The studies involving human participants were reviewed and approved by SingHealth Central Institutional Review Board, Singapore. For participants who were under 18 years old, written informed consent to participate in this study was provided by the participants’ legal guardian/next of kin. Written informed consent was obtained directly from participants if participants were over 18 years old.

## Author contributions

AB and VB performed the experiments. AB, S-YC, and SS contributed to manuscript preparation. GY and GT helped with primary tissue collection and processing prior to the culture experiments. SS conceived the study, supervised the experiments, obtained grant funding, and made the final decisions on the manuscript preparation. All authors contributed to the article and approved the submitted version.

## References

[1] Mead B Logan A Berry M Leadbeater W Scheven BA. Intravitreally transplanted dental pulp stem cells promote neuroprotection and axon regeneration of retinal ganglion cells after optic nerve injury. *Investig Ophthalmol Vis Sci.* (2013) 54:7544–56. 10.1167/iovs.13-13045 24150755

[2] Harper MM Grozdanic SD Blits B Kuehn MH Zamzow D Buss JE Transplantation of BDNF-secreting mesenchymal stem cells provides neuroprotection in chronically hypertensive rat eyes. *Investig Ophthalmol Vis Sci.* (2011) 52:4506–15. 10.1167/iovs.11-7346 21498611 PMC3175938

[3] Johnson TV Bull ND Martin KR. Identification of barriers to retinal engraftment of transplanted stem cells. *Investig Ophthalmol Vis Sci.* (2010) 51:960–70. 10.1167/iovs.09-3884 19850833 PMC2868445

[4] Tassoni A Gutteridge A Barber AC Osborne A Martin KR. Molecular mechanisms mediating retinal reactive gliosis following bone marrow mesenchymal stem cell transplantation. *Stem Cells.* (2015) 33:3006–16. 10.1002/stem.2095 26175331 PMC4832383

[5] Satarian L Nourinia R Safi S Kanavi MR Jarughi N Daftarian N Intravitreal injection of bone marrow mesenchymal stem cells in patients with advanced retinitis pigmentosa; A safety study. *J Ophthalmic Vis Res.* (2017) 12:58–64. 28299008 10.4103/2008-322X.200164 PMC5340065

[6] Oner A Gonen ZB Sinim N Cetin M Ozkul Y. Subretinal adipose tissue-derived mesenchymal stem cell implantation in advanced stage retinitis pigmentosa: a phase I clinical safety study. *Stem Cell Res Ther.* (2016) 7:178. 10.1186/s13287-016-0432-y 27906070 PMC5134260

[7] Kuriyan AE Albini TA Townsend JH Rodriguez M Pandya HK Leonard RE Vision loss after intravitreal injection of autologous “stem Cells” for AMD. *N Engl J Med.* (2017) 376:1047–53. 10.1056/NEJMoa1609583 28296617 PMC5551890

[8] Ho AC Chang TS Samuel M Williamson P Willenbucher RF Malone T. Experience with a subretinal cell-based therapy in patients with geographic atrophy secondary to age-related macular degeneration. *Am J Ophthalmol.* (2017) 179:67–80. 10.1016/j.ajo.2017.04.006 28435054

[9] Roozafzoon R Lashay A Vasei M Ai J Khoshzaban A Keshel SH Dental pulp stem cells differentiation into retinal ganglion-like cells in a three dimensional network. *Biochem Biophys Res Commun.* (2015) 457:154–60. 10.1016/j.bbrc.2014.12.069 25543058

[10] Gronthos S Mankani M Brahim J Robey PG Shi S. Postnatal human dental pulp stem cells (DPSCs) in vitro and in vivo. *Proc Natl Acad Sci USA.* (2000) 97:13625–30. 11087820 10.1073/pnas.240309797 PMC17626

[11] Mead B Logan A Berry M Leadbeater W Scheven BA. Concise review: dental pulp stem cells: a novel cell therapy for retinal and central nervous system repair. *Stem Cells.* (2017) 35:61–7. 10.1002/stem.2398 27273755

[12] Luo L He Y Wang X Key B Lee BH Li H Potential roles of dental pulp stem cells in neural regeneration and repair. *Stem Cells Int.* (2018) 2018:1731289. 10.1155/2018/1731289 PMC5964589 29853908

[13] Király M Porcsalmy B Pataki Á Kádár K Jelitai M Molnár B Simultaneous PKC and cAMP activation induces differentiation of human dental pulp stem cells into functionally active neurons. *Neurochem Int.* (2009) 55:323–32. 10.1016/j.neuint.2009.03.017 19576521

[14] Bray AF Cevallos RR Gazarian K Lamas M. Human dental pulp stem cells respond to cues from the rat retina and differentiate to express the retinal neuronal marker rhodopsin. *Neuroscience.* (2014) 280:142–55. 10.1016/j.neuroscience.2014.09.023 25242642

[15] Osakada F Jin ZB Hirami Y Ikeda H Danjyo T Watanabe K In vitro differentiation of retinal cells from human pluripotent stem cells by small-molecule induction. *J Cell Sci.* (2009) 122:3169–79. 19671662 10.1242/jcs.050393

[16] Bonnamain V Thinard R Sergent-Tanguy S Huet P Bienvenu G Naveilhan P Human dental pulp stem cells cultured in serum-free supplemented medium. *Front Physiol.* (2013) 4:357. 10.3389/fphys.2013.00357 24376422 PMC3858652

[17] Skog MS Nystedt J Korhonen M Anderson H Lehti TA Pajunen MI Expression of neural cell adhesion molecule and polysialic acid in human bone marrow-derived mesenchymal stromal cells. *Stem Cell Res Ther.* (2016) 7:113. 10.1186/s13287-016-0373-5 27528376 PMC4986182

[18] Ducret M Fabre H Degoul O Atzeni G McGuckin C Forraz N Immunophenotyping reveals the diversity of human dental pulp mesenchymal stromal cells in vivo and their evolution upon in vitro amplification. *Front Physiol.* (2016) 7:512. 10.3389/fphys.2016.00512 27877132 PMC5099238

[19] Tomlinson MJ Dennis C Yang XB Kirkham J. Tissue non-specific alkaline phosphatase production by human dental pulp stromal cells is enhanced by high density cell culture. *Cell Tissue Res.* (2015) 361:529–40. 10.1007/s00441-014-2106-3 25636587 PMC4529449

[20] Lackie PM Zuber C Roth J. Polysialic acid of the neural cell adhesion molecule (N-CAM) is widely expressed during organogenesis in mesodermal and endodermal derivatives. *Differentiation.* (1994) 57:119–31. 8070624 10.1046/j.1432-0436.1994.5720119.x

[21] Uezumi A Nakatani M Ikemoto-Uezumi M Yamamoto N Morita M Yamaguchi A Cell-Surface protein profiling identifies distinctive markers of progenitor cells in human skeletal muscle. *Stem Cell Rep.* (2016) 7:263–78. 10.1016/j.stemcr.2016.07.004 PMC4983081 27509136

[22] Sinanan ACM Hunt NP Lewis MP. Human adult craniofacial muscle-derived cells: neural-cell adhesion-molecule (NCAM; CD56)-expressing cells appear to contain multipotential stem cells. *Biotechnol Appl Biochem.* (2004) 40(Pt 1):25–34. 10.1042/BA20030185 15270704

[23] Battula VL Treml S Bareiss PM Gieseke F Roelofs H De Zwart P Isolation of functionally distinct mesenchymal stem cell subsets using antibodies against CD56, CD271, and mesenchymal stem cell antigen-1. *Haematologica.* (2009) 94:173–84. 10.3324/haematol.13740 19066333 PMC2635396

[24] Arthur A Rychkov G Shi S Koblar SA Gronthos S. Adult human dental pulp stem cells differentiate toward functionally active neurons under appropriate environmental cues. *Stem Cells.* (2008) 26:1787–95. 10.1634/stemcells.2007-0979 18499892

[25] Gronthos S Brahim J Li W Fisher LW Cherman N Boyde A Stem cell properties of human dental pulp stem cells. *J Dent Res.* (2002) 81:531–5. 12147742 10.1177/154405910208100806

[26] Chang CC Chang KC Tsai SJ Chang HH Lin CP. Neurogenic differentiation of dental pulp stem cells to neuron-like cells in dopaminergic and motor neuronal inductive media. *J Formos Med Assoc.* (2014) 113:956–65. 10.1016/j.jfma.2014.09.003 25438878

[27] Johnson TV Bull ND Hunt DP Marina N Tomarev SI Martin KR. Neuroprotective effects of intravitreal mesenchymal stem cell transplantation in experimental glaucoma. *Investig Ophthalmol Vis Sci.* (2010) 51:2051–9. 19933193 10.1167/iovs.09-4509 PMC2868400

[28] Jung J Kim JW Moon HJ Hong JY Hyun JK. Characterization of neurogenic potential of dental pulp stem cells cultured in xeno/serum-free condition: in vitro and in vivo assessment. *Stem Cells Int.* (2016) 2016:6921097. 10.1155/2016/6921097 27688776 PMC5027310

