# Supplementary material for: Dental pulp stem cells retain mesenchymal phenotype despite differentiation toward retinal neuronal fate in vitro
Source: Front Med (Lausanne). 2022 Oct 12;9:821361. doi: 10.3389/fmed.2022.821361 (PMC9596784; doi:10.3389/fmed.2022.821361)
Supplement: Supplementary file 1 [file Table_1.DOCX]

Supplementary Material

# Supplementary Tables

**Supplemental Table 1. List of antibodies used for flow cytometry and FACS analysis.**

| Antibody/ Probe | Fluorochrome | Vendor / Cat. No. / Clone |
| --- | --- | --- |
| Anti-human CD15 | FITC | BD Biosciences / 555401 / HI98 |
| Anti-human CD29 | PE | BD Biosciences / 556049 / HUTS-21 |
| Anti-human CD24 | PE | BD Biosciences / 555428 / ML5 |
| Anti-human CD24 | FITC | BD Biosciences / 560992 / ML5 |
| Anti-human CD90 | FITC | BD Biosciences / 561969 / 5E10 |
| Anti-human CD56 | PE | Life Technologies / MHCD5604 / MEM-188 |
| Anti-human CD34 | FITC | BD Biosciences / 560942 / My10 |
| Anti-human CD45 | FITC | BD Biosciences / 560976 / HI30 |

**Supplemental Table 2. Primers for Real-Time RT-PCR**

| Name |  | 5’ ⟵ Sequence ⟶ 3’ | Accession number |
| --- | --- | --- | --- |
| Oct3/4 | Forward | CCTGAAGCAGAAGAGGATCACC | NM_002701 |
|  | Reverse | AAAGCGGCAGATGGTCGTTTGG |  |
| Nanog | Forward | CTCCAACATCCTGAACCTCAGC | NM_024865 |
|  | Reverse | CGTCACACCATTGCTATTCTTCG |  |
| Sox2 | Forward | GCTACAGCATGATGCAGGACCA | NM_003106 |
|  | Reverse | TCTGCGAGCTGGTCATGGAGTT |  |
| Lhx2 | Forward | ACGCCAAGGACTTGAAGCAGCT | NM_004789 |
|  | Reverse | TTTCCTGCCGTAAGAGGTTGCG |  |
| Rax | Forward | ATCGTCAGACCGTTCCTCACGT | NM_032753 |
|  | Reverse | GCGACAGCCTCCTTGCTCAGA |  |
| Otx2 | Forward | GGAAGCACTGTTTGCCAAGACC | NM_021728 |
|  | Reverse | CTGTTGTTGGCGGCACTTAGCT |  |
| Pax6 | Forward | CTGAGGAATCAGAGAAGACAGGC | NM_001604 |
|  | Reverse | ATGGAGCCAGATGTGAAGGAGG |  |
| NGF | Forward | CCTCATCCCTGTCTATTGCTCC | NM_002507 |
|  | Reverse | GTTGGCTCCTTGCTTGTTCTGC |  |
| BDNF | Forward | CATCCGAGGACAAGGTGGCTTG | NM_170734 |
|  | Reverse | GCCGAACTTTCTGGTCCTCATC |  |
| NTF3 | Forward | CAAGCAGATGGTGGACGTTAAGG | NM_002527 |
|  | Reverse | TCGCAGCAGTTCGGTGTCCATT |  |
| GDNF | Forward | CGCCGAAGACCGCTCCCTCG | NM_000514 |
|  | Reverse | ATCCATGACATCATCGAACTGATC |  |
| GFAP | Forward | CTGGAGAGGAAGATTGAGTCGC | NM_002055 |
|  | Reverse | ACGTCAAGCTCCACATGGACCT |  |
| CD56 | Forward | CATCACCTGGAGGACTTCTACC | NM_181351 |
|  | Reverse | CAGTGTACTGGATGCTCTTCAGG |  |
| Glast | Forward | GGTTGCTGCAAGCACTCATCAC | NM_004172 |
|  | Reverse | CACGCCATTGTTCTCTTCCAGG |  |
| NeuroD1 | Forward | GGTGCCTTGCTATTCTAAGACGC | NM_002500 |
|  | Reverse | GCAAAGCGTCTGAACGAAGGAG |  |
| MITF | Forward | GGCTTGATGGATCCTGCTTTGC | NM_198159 |
|  | Reverse | GAAGGTTGGCTGGACAGGAGTT |  |
| CHX10 | Forward | GGAGAAGGCATTCAACGAAGCC | NM_182894 |
|  | Reverse | ACTTGGCTCGACGGTTCTGGAA |  |
| RHO | Forward | AGCTCGTCTTCACCGTCAAGGA | NM_000539 |
|  | Reverse | CCAGCAGATCAGGAAAGCGATG |  |
| PEDF | Forward | TGAAGGCGAAGTCACCAAGTCC | NM_002615 |
|  | Reverse | CCATCCTCGTTCCACTCAAAGC |  |
| CD29 | Forward | GGATTCTCCAGAAGGTGGTTTCG | NM_033667 |
|  | Reverse | TGCCACCAAGTTTCCCATCTCC |  |
| CD90 | Forward | GAAGGTCCTCTACTTATCCGCC | NM_006288 |
|  | Reverse | TGATGCCCTCACACTTGACCAG |  |
