# Supplementary material for: Dental pulp stem cells retain mesenchymal phenotype despite differentiation toward retinal neuronal fate in vitro
Source: Front Med (Lausanne). 2022 Oct 12;9:821361. doi: 10.3389/fmed.2022.821361 (PMC9596784; doi:10.3389/fmed.2022.821361)
Supplement: Supplementary file 2 [file Table_2.DOCX]

Supplementary figure 2

Immunostaining images (such as shown in Fig 6) were used to perform quantitation of the percentage of cells double positive for CD90 with the respective retinal marker. Since CD90 is a cell surface marker and all three retinal markers used here were nuclear markers ( OTX2, RAX and MITF), cell permeabilization was required to elicit proper signal of the latter. This resulted in considerable loss of CD90 signal. This was adjusted for in control images when using Image J to designate CD90 positivity and the thresholds then applied to the test images. The results shown above are from 3 separate experiments with n>50 cells in each group.
