# Supplementary figures and images for: Dental pulp stem cells retain mesenchymal phenotype despite differentiation toward retinal neuronal fate in vitro
Source: Front Med (Lausanne). 2022 Oct 12;9:821361. doi: 10.3389/fmed.2022.821361 (PMC9596784; doi:10.3389/fmed.2022.821361)

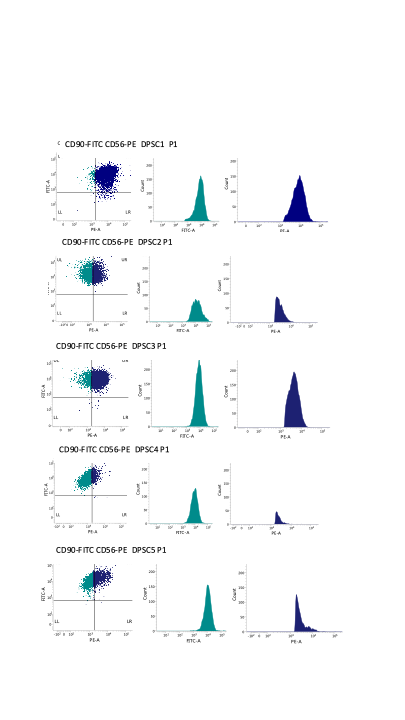

Supplement: Supplementary file 3 [file Image_1.TIFF]
